# New and Stale Bread

**Published:** 1883-07

**Authors:** 


### NEW AND STALE BREAD.

A celebrated French chemist, M. Boussingault, has recently been engaged investigating the nature of the change which takes place when bread becomes stale, something which has hitherto not been understood. In the course of his experiments a circular loaf, 12 inches in diameter and 6 inches thick, was taken from an oven heated to 240° Reaumur, and a thermometer forced into it three inches. The thermometer indicated 78° R. (207.5 F.) The loaf was then taken to a room the temperature of which was 15° R. (66° F.), and temperature of loaf sunk to 19° R. (73° F.), and in 24 hours to 15° (66° F.), and in 36 hours to 14° (63.5). In the first 48 hours it lost only two ounces in weight. After six days the loaf was again put in the oven, and when the thermometer had indicated its temperature had risen to 55° R. (156° F.), it was cut open and found to be fresh, and to possess the same qualities as if it had been taken out of the oven the first time ; but it had lost 12 ounces in weight. Experiments were made with slices of bread with similar results, proving conclusively that new bread differs from the old, not by containing a larger proportion of water, but by a peculiar molecular condition. This commences and continues to change during cooling, but by again heating the bread to a certain temperature, it is restored to its original state. It is the mechanical state which makes new bread less digestible than old. The former is soft, elastic and glutinous in all its parts, and ordinary mastication fails to reduce it to a sufficiently digestible condition.

